# Opportunistic Water-Borne Human Pathogenic Filamentous Fungi Unreported from Food

**DOI:** 10.3390/microorganisms6030079

**Published:** 2018-08-03

**Authors:** Monika Novak Babič, Jerneja Zupančič, João Brandão, Nina Gunde-Cimerman

**Affiliations:** 1 Biotechnical Faculty, University of Ljubljana, Jamnikarjeva 101, SI-1000 Ljubljana, Slovenia; jerneja.zupancic@bf.uni-lj.si (J.Z.); nina.gunde-cimerman@bf.uni-lj.si (N.G.-C.); 2 Department of Environmental Health, National Institute of Health Doutor Ricardo Jorge, Av. Padre Cruz, 1649-016 Lisboa, Portugal; joao.brandao@insa.min-saude.pt

**Keywords:** exposure, filamentous fungi, opportunistic infections, water

## Abstract

Clean drinking water and sanitation are fundamental human rights recognized by the United Nations (UN) General Assembly and the Human Rights Council in 2010 (Resolution 64/292). In modern societies, water is not related only to drinking, it is also widely used for personal and home hygiene, and leisure. Ongoing human population and subsequent environmental stressors challenge the current standards on safe drinking and recreational water, requiring regular updating. Also, a changing Earth and its increasingly frequent extreme weather events and climatic changes underpin the necessity to adjust regulation to a risk-based approach. Although fungi were never introduced to water quality regulations, the incidence of fungal infections worldwide is growing, and changes in antimicrobial resistance patterns are taking place. The presence of fungi in different types of water has been thoroughly investigated during the past 30 years only in Europe, and more than 400 different species were reported from ground-, surface-, and tap-water. The most frequently reported fungi, however, were not waterborne, but are frequently related to soil, air, and food. This review focuses on waterborne filamentous fungi, unreported from food, that offer a pathogenic potential.

## 1. Water in Natural and Anthropogenic Environments

Fresh water, one of our essential needs, is valuable not only for drinking, but also as one of the driving forces for the development of humankind [1]. As such, it must be accessible and safe, especially in overpopulated areas, where pollution occurs more frequently [2]. Fresh water may be divided depending on the location, flow, and anthropogenic influence into different types: groundwater, surface water (streams, rivers, lakes), and tap-water [1,3,4]. Influenced by specific physio-chemical characteristics, their microbial populations differ [4]. The most important natural and anthropogenic abiotic and biotic factors influencing microbial presence in different water sources, include location of the main water source, water flow, ion composition, presence of organic matter, pollution rate, and water cleaning processes (Table 1) [1,3]. The latter are particularly important to maintain safe drinking and recreational water, preventing outbreaks of (mainly) gastrointestinal illnesses or irritation due to chemicals [2]. Chemical and microbiological parameters are listed in regulations covering quality of different water sources depending on water usage [1]. In Europe, the main documents regulating water quality are the Drinking Water Directive (98/83/CE), the Directive 2009/54/EC on mineral water and spring water, and Council Directive 76/160/EEC for bathing water. Whilst these documents successfully control and prevent the spreading of gastrointestinal illnesses (mainly attributed to bacteria and viruses), they leave out monitoring of causative agents of opportunistic fungal infections [1]. Only in Europe, over the last 30 years, more than 400 different fungal species have been reported from groundwater, surface water, and drinking water; among which 46 were classified as Biosafety Level 2 [1].

However, the majority of reported fungal species were not related exclusively to water, but they can be found also in air, soil, and food [1,5]. Food-related opportunistic filamentous fungi, isolated also from water, included those from the genera *Acremonium*, *Alternaria*, *Aspergillus*, *Chaetomium*, *Fusarium*, *Mucor*, *Lichtheimia*, *Paecilomyces*, *Penicillium*, *Phoma*, *Scopulariopsis*, and *Trichoderma* [1,5]. Paterson and Lima (2017) discussed their presence on food, and their medical relevance, in detail [5]. The present paper thus focuses only on opportunistic filamentous fungi related to water, and not reported from food [5].

## 2. Water-Related Filamentous Fungi as Causative Agents of Opportunistic Infections

Different types of water carry diverse fungal biota, but some species are more likely connected to the specific water type. For instance, surface water sources harbor fungi that are mainly associated with plants and their debris, and causative agents of plant diseases, such as representatives from *Botrytis* and *Cylindrocarpon* [1,3]. Similarly, melanised fungi from *Aureobasidium*, *Exophiala*, and *Rhinocladiella* are mainly associated with groundwater [1,6]. Knowing the origin of waterborne opportunistic fungi is of a great importance for health risk assessments. Reports related to fungal diseases are numerous, primarily due to the increasing number of transitory and serious immune alterations, particularly among people who spend a long time in hospital or other healthcare facilities [7,8]. Reports include allergies, opportunistic infections and intoxications [7,8]. Skin-related diseases, however, are the most common outcome of fungal infections, with an estimated number of patients on a global scale of over one billion [9]. Infections with filamentous fungi from water may occur in different ways—via exposure during sport and recreation, drinking, and personal and home hygiene, including aerosol intake from breathing during showering [1].

The present work focuses on reports on filamentous fungi with opportunistic potential isolated from water during the last five years. The most abundant waterborne filamentous fungi belong to the phylum Ascomycota, followed by species from the phylum Mucoromycota. Basidiomycota phylum does not contain exclusively waterborne species of filamentous fungi [10].

### 2.1. Filamentous Opportunistic Fungi with Possible Groundwater Origin

Groundwater is rarely directly associated with fungal diseases, due to its restricted public access, but it serves as a vector for fungal cells and spores during the preparation of groundwater-derived tap-water. The presence of fungi in groundwater can thus affect the tap-water quality and this poses a health risk during tap-water-related activities [1,6,11,12] (Table 2). As reported by Novak Babič et al. (2017), groundwater derived tap-water will more likely harbor species from *Verticillium* and different melanised fungi. Among them, fungi from *Cyphellophora* (former members of *Phialophora*) and *Exophiala* were the most commonly encountered [1] (Table 2). All currently known species within *Verticillium* (Plectosphaerellaceae) are opportunistic or true plant pathogens, and despite being commonly detected in water, they do not present a significant health risk for humans [13]. Melanised fungi from *Cyphellophora* (Cyphellophoraceae) have a more diverse ecology. They have been linked to humid indoor niches, swimming facilities, but also to plant and human diseases. They mainly cause surface infections of skin and nails [14,15] (Table 2). Yet, *Exophiala* (Herpotrichiellaceae) includes many water-related opportunistic pathogens, most of them classified under Biosafety Level 2 [8,15]. Recent literature reported on the presence of *E. castellanii*, *E. dermatitidis*, *E. jeanselmei*, *E. oligosperma*, *E. phaeomuriformis* and *E. spinifera* mainly from groundwater and groundwater derived tap-water, pointing towards groundwater as a possible source of these black fungi [6,12,16,17] (Table 2). *Exophiala* are associated with diverse spectra of opportunistic diseases such as otitis, keratitis, phaeohyphomycosis, and respiratory, cutaneous, and subcutaneous infections. Although rare, disseminated, systemic, and cerebral infections can be fatal [8].

### 2.2. Filamentous Opportunistic Fungi with Possible Origin from Surface Water

Surface water include streams, rivers, and lakes that can be used for preparation of drinking water, but they may also serve recreational purposes [26,40,41]. During recreational activities, people are exposed to fungi via direct skin contact and through inhalation of aerosols [1]. The latest literature links surface water with the presence of ascomycetous species of *Cylindrocarpon*, *Microsporum*, *Phialemonium*, *Rhinocladiella*, and fungi from subphylum Mucoromycotina (Table 2) [19,26,35]. *Cylindrocarpon* (Nectriaceae) species are closely related to *Fusarium* and *Nectria*, and are causative agents of plant and trees diseases [42]. Human-related infections were rarely reported and they usually occur after trauma. They include mycetoma, cutaneous infections, and keratitis [8]. Species of *Phialemonium* (Cephalothecaceae) are commonly found in air, soil, and polluted or industrial water [43], but have also been detected in drinking water (Table 2). Their presence in tap-water could have a surface water origin. They are opportunistic pathogens, reported to cause endocarditis, keratitis, and peritonitis, respiratory, subcutaneous, and systemic infections [8]. Also close relatives of *Exophiala*, black filamentous fungi *Rhinocladiella* (Herpotrichiellaceae) were isolated from both groundwater and surface water, and they are one of the common contaminants of drinking water [6,12,17,26,33] (Table 2). They can cause chromoblastomycosis and cutaneous infections, particularly in tropical and sub-tropical regions [8]. Surface water can be a possible vector for the transmission of dermatophytes *Microsporum* (Arthrodermataceae) [27,28,29]. *Microsporum* species are causative agents of different tineas (Table 2) and they are highly transmittable between animal hosts and people [8]. The highest degree of infections has been reported for children under the age of nine [8]. Also, *Cunninghamella* (Cunninghamellaceae), *Rhizopus* (Rhizopodaceae), and *Rhizomucor* (Lichtheimiaceae) species, classified in subphylum Mucoromycotina, are commonly associated with surface water (Table 2), due to their ability to degrade plants and debris [44]. Although members of these genera can additionally cause diseases in insects, they may colonize also other animals and humans. According to the revised taxonomy, fungi causing mucormycosis are classified in the phylum Mucoromycota, and subphylum Mucoromycotina. Representative taxa within *Rhizopus*, *Mucor*, *Lichtheimia* (formerly *Absidia*), *Cunninghamella*, *Rhizomucor*, and *Saksenaea* constitute those that are identified as causative agents of the majority of cases of mucormycosis [45]. Approximately half of all mucormycosis cases are caused by *Rhizopus* spp. [46]. Mucormycoses in humans are limited to severely immuno-compromised people, those with diabetes mellitus, or those after experiencing trauma [44]. Clinical manifestations include disseminated, cutaneous, subcutaneous, respiratory, and rhinocerebral infections (Table 2) [8].

One of the most serious fungal infections of the lungs and brain that may afflict patients follows exposure by near–drowning events due to inhalation of contaminated water [47,48]. The most common fungi associated with near-drowning syndrome belong to the *Scedosporium apiospermum* complex (Microascaceae), including fungi *Pseudallescheria* and its anamorph *Scedosporium* [21]. The disease develops slowly, and only after several weeks or months do cerebral abscesses or pulmonary infection appear, often with a fatal outcome [47,48]. Fungi from *Scedosporium apiospermum* complex were also related to otitis, subcutaneous, and disseminated infections (Table 2) [8]. Another accidental exposure to fungi originating from surface water can occur after natural disasters, like floods. *Stachybotrys chartarum* and *S. ramosus* (Stachybotryaceae) are particularly associated with sick building syndrome, often developed in such instances [19,34]. Due to the high number of spores on water-damaged buildings [36,37], long term exposure to species from *Stachybotrys* may lead to respiratory and cutaneous infections, hemorrhage, irritation, and allergies [8,36].

### 2.3. Filamentous Opportunistic Fungi from Tap Water Colonise Water-Related Indoor Niches and Recreational Facilities

In spite of well-established water cleaning processes, fungi originating from groundwater or surface water enter water distribution systems and form stable biofilms on different pipe materials [49]. Case reports about fungal contamination of tap-water and biofilm establishment in hospitals and other healthcare facilities have been published. Fungal propagation, biofilm formation, and consequent infections were common at dental, hemodialysis, pediatric, and intensive care units [50,51,52,53].

There is little data on the health effect of fungi after drinking contaminated water [1]. However, modern society uses substantial amounts of tap-water for showering, bathing, cooking, and cleaning [1,54]; most frequently performed with household appliances, such as washing machines and dishwashers. They represent novel niches for propagation of waterborne fungi, particularly polyextremotolerant species *Exophiala dermatitidis* and *E. phaeomuriformis*, and these should be taken into consideration for risk assessment linked to fungal diseases [15,17,55]. Together with the above-mentioned fungi, *Phialophora* (Herpotrichiellaceae) and *Ochroconis* (Sympoventuriaceae) were recently linked to nutrient-poor tap-water [30,31] and indoor wet niches, such as bathrooms and washing machines [15,55]. Some species within *Phialophora* and *Ochroconis* were reported as causative agents of cutaneous and subcutaneous infections (Table 2) [8].

Tap water is used for recreational purposes in swimming pools, jacuzzies, baths, and saunas [22]. Despite the addition of chlorine-based chemicals, a variety of waterborne fungi were isolated [22,56]. The most common diseases reported to develop after visits to recreational public facilities, are Tineas and other cutaneous infections. Tineas are commonly caused by dermatophytes *Trichophyton* (Arthrodermataceae). *Trichophyton mentagrophytes*, *T. schoenleinii*, *T. tonsurans*, *T. verrucosum*, and *T. violaceum*, amongst others, were recently isolated from tap-water, and are related to swimming pools (Table 2) [8,22,27,35].

## 3. Conclusions

Due to the expanding human population, safe drinking and recreational waters remains one of the most important present and future goals. Although fungi are not mentioned in the present regulations that define water quality, they are constantly isolated from water microbial communities. Reported waterborne filamentous fungi that are associated with opportunistic infections belonged to 12 families and 16 genera from the phyla Ascomycota and Mucoromycota. Human opportunistic fungi, sometimes also linked to plants and insect diseases, were more likely isolated from surface water, while melanised fungi dominated groundwater and groundwater-derived tap-water. Water-borne filamentous fungi were commonly reported to be causative agents of cutaneous, subcutaneous, and respiratory infections among people with transitory and serious immune alterations. Case reports were mainly related to immuno-compromised people who were held in hospitals. In the future, the incidence of fungal infections could be minimized with the inclusion of fungal parameters in water monitoring.

## Figures and Tables

**Table 1:** Natural and anthropogenic biotic and abiotic factors, influencing microbial presence in different water sources.

Biotic and Abiotic Factors	Water Source					
	Natural Environment				Human-Made Environment	
	Groundwater	Rivers & Streams	Lakes	Mineral Water	Tap Water	Water from Swimming Facilities
Primary water source location	Underground	Surface	Surface	Underground/Surface	Underground/Surface	Underground/Surface
Water flow	Stable	Variable	Stable	Stable/Variable	Variable	Stable/Variable
Sun irradiation	Absent	Present	Present	Absent/Present	Absent	Absent/Present
Temperature	Stable	Variable	Variable	Stable	Variable	Stable
Ion composition	Stable	Variable	Variable	Stable	Stable	Variable
pH	Stable	Variable	Variable	Stable	Stable	Stable
Organic matter concentration	Low	High	High	Low/High	Low	Low
Dissolved oxygen concentration	Low	Low/High	Low	Low/High	High	High
Water treatment	Absent	Absent	Absent	Absent/Ozonation	Aeration Ultrafiltration UV-treatment Ozonation Chlorination	Additional chlorination
Effect of materials in distribution systems	Absent	Absent	Absent	Absent	Present	Present

**Table 2:** Opportunistic waterborne filamentous fungi not reported from food, and their effects on human health.

Fungal Species	BSL *	Water Type	Effect on Health	References
**Phylum Ascomycota**				
*Cylindrocarpon aquaticum*	1	Surface water	No data	[18,19]
*Cylindrocarpon* sp.	1/2	Surface water	Cutaneous infections Keratitis Mycetoma	[8,19,20]
*Cyphellophora europaea*	2	Surface water Tap water	Cutaneous infections	[6,8,21]
*Cyphellophora oxyspora*	1	Swimming pool	Cutaneous infections	[8,22]
*Cyphellophora reptans*	1	Groundwater Tap water	Superficial infections	[6,8,23]
*Cyphellophora sessilis*	1	Groundwater Tap water	No data	[6]
*Exophiala castellanii*	2	Groundwater Tap water	Cutaneous infections	[6,8,11,16,23]
*Exophiala dermatitidis*	2	Glacier water Mineral water Groundwater Tap water	Cerebral infections Cutaneous infections Disseminated infections Keratitis Otitis Respiratory infections Subcutaneous infections	[8,12,17,24]
*Exophiala jeanselmei*	2	Tap water	Cutaneous infections Subcutaneous infections	[8,11,16]
*Exophiala oligosperma*	2	Groundwater Surface water Tap water	Cerebral infections Cutaneous infections Onychomycosis Subcutaneous infections	[6,8,12,25,26]
*Exophiala phaeomuriformis*	2	Tap water	Cutaneous infections Endocarditis Subcutaneous infections	[6,8,12,17]
*Exophiala spinifera*	2	Surface water Tap water Bottled water	Cutaneous infections Disseminated infections Sinusitis Subcutaneous infections	[8,11,16,21]
*Microsporum canis*	2	Surface water	Tinea capitis Tinea corporis	[8,27]
*Microsporum gypseum*	1	Surface water	Tinea corporis Tinea faciei Tinea manus	[27,28]
*Microsporum* sp.	1/2	Surface water	Different Tineas	[8,29]
*Ochroconis bacilliformis*	1	Tap water	No data	[30]
*Ochroconis constricta*	1	Tap water	No data	[30]
*Ochroconis globalis*	1	Tap water	No data	[30,31]
*Ochroconis musae*	1	Tap water	Cutaneous infections Subcutaneous infections	[6,8,30]
*Ochroconis tshamytschae*	1	Tap water	Cutaneous infections Subcutaneous infections	[8,30,32]
*Phialophora bubakii*	1	Tap water	Subcutaneous infections	[8,33]
*Phialemonium obovatum*	2	Tap water	Endocarditis Keratitis Peritonitis Subcutaneous infections Systemic infections	[8,33]
*Phialemonium* sp.	1/2	Surface water Tap water	Respiratory infections Subcutaneous infections Systemic infections	[8,34,35]
*Rhinocladiella aquaspersa*	2	Surface water Tap water	Chromoblastomycosis	[8,26,33]
*Rhinocladiella similis*	2	Groundwater Surface water Tap water	Cutaneous infections	[6,8,12,17,26]
*Scedosporium apiospermum* complex (including former *Pseudallescheria boydii*)	2	Surface water Tap water	Cerebral infections Respiratory infections Subcutaneous infections Systemic infections	[8,21]
*Scedosporium prolificans*	2	Tap water	Disseminated infections Respiratory infections Otitis	[8,35]
*Stachybotrys chartarum*	1	Surface water	Cutaneous infections Respiratory infections	[19,34,36]
*Stachybotrys ramosus*	1	Surface water	Cutaneous infections Respiratory infections	[18,37]
*Trichophyton mentagrophytes*	2	Tap water Swimming pool	Different Tineas	[8,22,35]
*Trichophyton schoenleinii*	2	Tap water	Tinea capitis Tinea corporis	[8,35]
*Trichophyton tonsurans*	2	Surface water	Tinea capitis Tinea corporis	[8,27]
*Trichophyton verrucosum*	2	Tap water	Onychomycosis Tinea barbae	[8,35]
*Trichophyton violaceum*	2	Tap water	Tinea capitis Tinea corporis	[8,35]
*Verticillium* spp.	1	Groundwater Tap water	Keratitis	[34,35]
**Phylum Mucoromycota**				
*Cunninghamella elegans*	1	Surface water	Respiratory infections	[8,38]
*Cunninghamella* sp.	1/2	Tap water	Cutaneous infections Respiratory infections Rhinocerebral infections	[8,34]
*Rhizopus* spp.	1	Surface water	Cutaneous infections Respiratory infections Rhinocerebral infections Subcutaneous infections	[8,21]
*Rhizopus stolonifer*	1	Surface water	Rhinocerebral infections	[8,27]
*Rhizomucor* spp.	1/2	Surface water Tap water	Disseminated infections Systemic infections	[8,35,39]

* Biosafety Level.
